# Review of the Umthombo Youth Development Foundation scholarship scheme, 1999–2013

**DOI:** 10.4102/phcfm.v7i1.739

**Published:** 2015-03-31

**Authors:** Andrew Ross, Gavin MacGregor, Laura Campbell

**Affiliations:** 1 Department of Family Medicine, University of KwaZulu-Natal, South Africa; 2 Umthombo Youth Development Foundation, South Africa

## Abstract

**Introduction:**

Staffing of rural and remote facilities is a challenge throughout the world. Umthombo Youth Development Foundation (UYDF) has been running a rurally based scholarship scheme since 1999. The aim of this review is to present data on the number of students selected, their progress, graduation and work placement from inception of the scheme until 2013.

**Methods:**

Data were extracted from the UYDF data base using a data collection template to ensure all important information was captured.

**Results:**

Since 1999, 430 rural students across 15 health disciplines have been supported by UYDF. The annual pass rate has been greater than 89%, and less than 10% of students have been excluded from university. All graduates have spent time working in rural areas (excluding the 32 currently doing internships) and 72% (52/73) of those with no work-back obligation continue to work in rural areas.

**Discussion and conclusion:**

The UYDF model is built around local selection, compulsory academic and peer mentoring and social support, comprehensive financial support and experiential holiday work. The results are encouraging and highlight the fact that rural students can succeed at university and will come back and work in rural areas. With 46% of the South African population situated rurally, greater thought and effort must be put into the recruitment and training of rural scholars as a possible solution to the staffing of rural healthcare facilities. The UYDF provides a model which could be replicated in other parts of South Africa.

## Abstrait

**Examen du Programme de Bourses de la Fondation Umthombo pour le Développement de la Jeunesse, 1999–2013.**

**Introduction:**

La dotation en personnel des institutions en zones rurales et lointaines est un défi dans le monde entier. La Fondation Umthombo pour le Développement de la Jeunesse (UYDF) a organisé un programme de bourses en milieu rural depuis 1999. Le but de cet examen est de fournir des données sur le nombre d’étudiants sélectionnés, leurs progrès, l'obtention de leur diplôme et leur placement professionnel depuis le début du programme jusqu’à 2013.

**Méthodes:**

Les données proviennent de la base de données de l'UYDF en se servant d'un modèle de collecte de données afin que toutes les informations importantes soient saisies.

**Résultats:**

Depuis 1999, 430 étudiants ruraux dans 15 disciplines de la santé ont été aidés par l'UYDF. Le taux de réussite annuel était de plus de 89%, et moins de 10% des étudiants ont été exclus de l'université. Tous les diplômés ont travaillé en zone rurale (à l'exception des 32 qui sont en train de faire leur stage) et 72% (52/73) de ceux qui n'ont pas à rembourser leur bourse par le travail continuent à travailler dans les zones rurales.

**Discussion et conclusion:**

Le modèle de l'UYDF s'articule autour de la sélection locale, du mentorat académique et par les pairs, et du soutien social, un soutient financier complet et du travail expérientiel de vacances. Les résultats sont encourageants et montrent que les étudiants ruraux peuvent réussir à l'université et reviendront travailler dans les zones rurales. Avec 46% de la population sud-africaine vivant à la campagne, il faut s'efforcer de recruter et de former d'avantage d’étudiants ruraux comme solution possible pour pourvoir en personnel les services de santé ruraux. L'UYDF fournit un modèle qui pourrait être reproduit dans d'autres parties de l'Afrique du Sud.

## Background

Staffing of rural and remote health facilities is a challenge throughout the world. The World Health Organization (WHO) estimates that there is a global shortage of 4.3 million doctors and nurses, with up to 1 billion people without access to healthcare workers.^1^ The Department of Health in its *Human Resources for Health for South Africa 2030* estimated that there is a shortage of 14 932 professional nurses, 4145 doctors, 778 pharmacists, 1777 social workers and 345 physiotherapist in South Africa (SA), with rural areas impacted by staff shortages more than urban areas.^2^

In Australia, Canada and the United States of America, recruiting rural origin scholars who return to work in rural areas after training has been shown to be an effective strategy for increasing staffing levels at rural and remote facilities.^3,4^ Using the pipeline metaphor, various strategies have been employed to increase the number of rural origin students in these countries. These include the promotion of careers in medicine at high schools, reservation of a certain percentage of places for rural origin students at medical schools, increasing the rural content and exposure to rural medicine at university, and recently the development of rurally located medical schools.^5^

Although there is substantial evidence from First World countries, there is relatively little evidence from developing countries that rural recruitment impacts on staffing levels at rural facilities. In 2003 a South African study reviewed where 138 rural origin and 140 urban origin students where working as health care professionals (HCPs). This study concluded that rural students were more likely to work in rural areas than urban origin students (38% vs. 12% respectively).^6^ In 2003 Ross and Reid reviewed a number of HCPs who remained at a rural district hospital (DH) post-community service and found that the numbers who remained were small (22/278; 8%). The authors concluded that rural origin HCPs and those with provincial work-back obligations were more like to stay at a rural DH than those who grew up in an urban area or those without any contractual obligations.^7^

Currently there appear to be no systematic efforts to promote health science careers in rural areas of SA, and dysfunctional schools make entry into and success at medical school a challenge.^8^ Most South African healthcare training institutions currently have a race-based admission policy to address the imbalances of the apartheid past. The University of KwaZulu-Natal has recently introduced a policy of admitting 28% quintile 1 and 2 students to the medical school to increase representation from these schools. Although many rural schools may be represented by quintile 1 and 2 schools this is an incidental consideration and not a policy to ensure adequate selection of rural origin students. Tumbo's 2009 study, which looked at rural representation at the nine health education facilities in SA, showed that rural origin students accounted for 27.4%, 22.4%, 26.7%, and 24.8% in medicine, physiotherapy, occupational therapy and dentistry respectively – significantly lower than the national rural population ratio.^9^ As such, the recruitment of rural origin students could be considered to be an issue of social justice, important for the provision of health services in rural areas.

With 46% of the South African population situated rurally,^10^ it would appear that geographical origin must become an important selection consideration at health training institutions. Greater effort should be put into the recruitment of rural scholars, the prioritisation of places for them to study at medical school and other health training institutions, provision of adequate support to enable them to succeed, as well as creation of posts, and other postgraduate career opportunities in rural areas.

Umthombo Youth Development Foundation (UYDF) has been running a rurally based scholarship scheme since 1999. The scholarships scheme was initially established to address staff shortages at a rural DH. The conceptualisation of the scheme was based on evidence from studies in Australia and Canada which showed that rural origin students are more likely to work in rural areas than urban origin students.^11,12^ UYDF students are selected by the local hospital selection committee and sign a year-for-year work-back contract with UYDF. UYDF provides comprehensive financial support at university and a compulsory structured peer, academic and social mentoring programme. The hospital provides opportunities for experiential holiday work experience and employment opportunities on completion of their degree. By any definition (geography, work opportunities, distance, population density, etc.) the students supported by UYDF would be considered to be rural.^13^

### Aim and objective

The aim of this review is to present data on the number of students selected, their progress, graduation and work placement from inception of the UYDF scheme in 1999 until 2013. It is hoped that this review will stimulate debate on admission policies and indicate possible solutions to the staffing challenges at rural healthcare facilities.

## Methods

Data were extracted from the UYDF data base using a data collection template to ensure that all important information was captured. All students supported by UYDF have been included (even those partially funded by UYDF and who received a provincial bursary or other funding during the course of their studies). Graduates were contacted by UYDF staff to verify the information available at the office and to obtain any outstanding information.

### Ethical considerations

Ethical permission for this study was given by the Human and Social Science Ethics Committee of the University of KwaZulu-Natal (HSS/0228/014)

## Results

**FIGURE 1:**
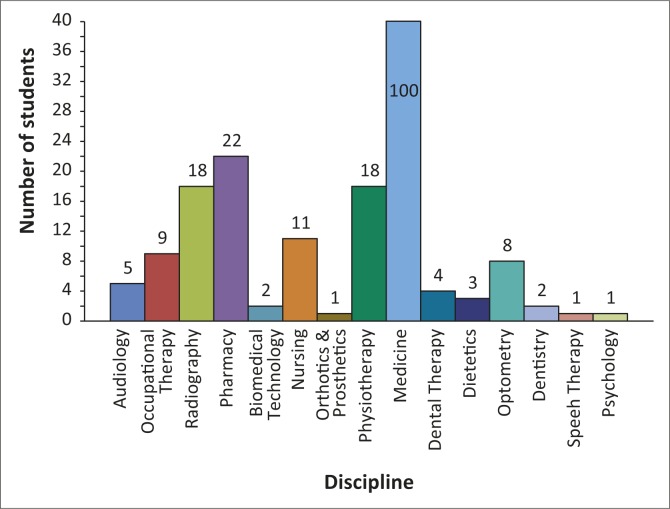
Current students by discipline (*n* = 205); there are 100 medical students (off the scale).

Some health science courses are three years in duration (dental therapy, environmental health, radiography, biomedical technology), some four years (physiotherapy, pharmacy, nursing) and others six years (medicine). This means that intake rates do not necessarily correspond with graduation rates; see Table 1 for details. In 2007 UYDF moved from a voluntary run organisation to having full-time staff members (Director, full-time student mentor and administrative support), and the dramatic increase in the total number of students supported since 2008 can be attributed to this. Currently UYDF is supporting 205 students across 15 health disciplines, 65% of whom are women. A breakdown of students by discipline is presented in Figure 1.

**TABLE 1:** Student numbers supported by UYDF since 1999.

Year	New students	Cumulative total*	No. passed**	Number repeating	Number excluded	Number graduated	Pass rate (%)
1999	4	4	4	0	0	0	100
2000	5	9	8	1	0	0	89
2001	6	15	13	1	1	0	87
2002	22	36	29	1	6	2	81
2003	17	45	40	4	1	7	88
2004	12	49	47	2	0	5	96
2005	12	56	50	3	3	10	89
2006	11	54	47	4	3	12	87
2007	17	56	52	0	4	13	93
2008	26	65	53	10	2	7	82
2009	37	83	71	10	2	19	86
2010	36	108	97	9	2	15	90
2011	61	152	132	16	2	25	87
2012	56	181	166	15	8 + 2 (poor attitudes)	22	92
2013	42	191	179	8	4 + 1 (ill health)	48	94
2014	66	205	-	-		-	-
Total	-	430	-	-	41 (9.5%)	185	-

*, Cumulative total = new students + existing students – number graduated – number excluded.

**, Number passed = number of students who were able to progress (they may have been carrying some subjects).

**FIGURE 2:**
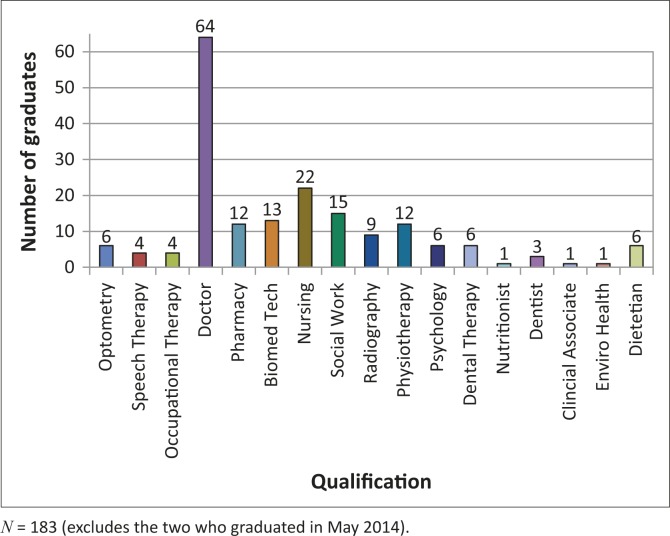
Breakdown of graduates by qualification.

As of May 2014 there are 185 UYDF graduates. A breakdown of graduates by discipline is presented in Figure 2.

**FIGURE 3:**
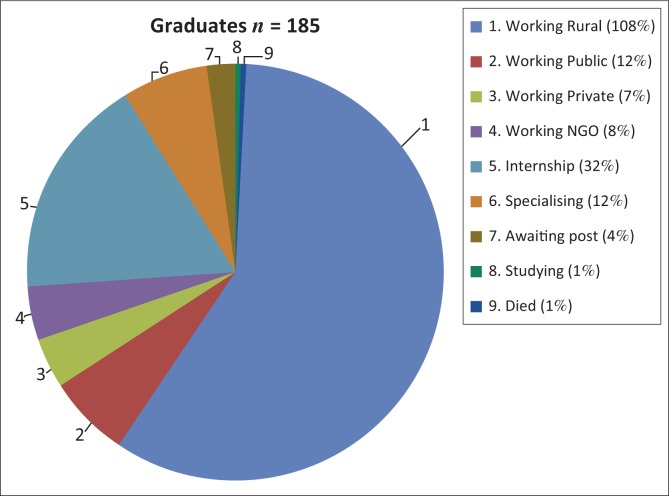
Current graduates by current place of work.

Figure 3 shows the breakdown of graduates by current place of work

Some public sector hospitals are in urban areas, but most of the non-governmental organisations (NGOs) and some private practices where graduates work are in rural areas. Not all students are supported by UYDF for the duration of their training, as some students obtain a provincial bursary during the course of their studies. The provincial bursary programme has different work-back obligations; their graduates may work in any KwaZulu-Natal provincial hospital (including urban hospitals), and may engage in postgraduate training during their work-back obligation.

**FIGURE 4:**
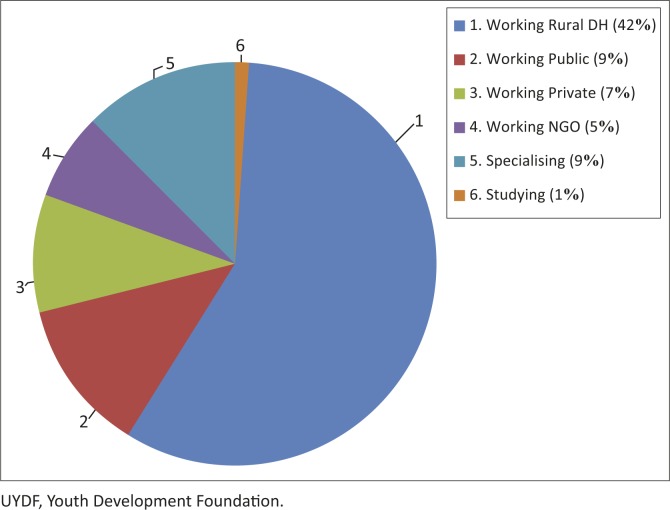
Work placement of graduates with no work-back obligation to UYDF (*n* = 73).

All graduates who received any support from UYDF have spent at least a year working at a rural DH (this excludes the 32 currently doing their internship, as it is not possible to do ones internship at a rural DH in SA). Nine graduates have bought themselves out of a portion of their UYDF contract, and only one has defaulted on their contractual obligations. Of the 73 graduates who have completed their contractual obligations to UYDF, 52 (71%) continue to practice in a rural area, 42 in rural DHs, and the balance are working for rural NGOs or in rural private practice. A breakdown of these graduates by location is presented in Figure 4.

**FIGURE 5:**
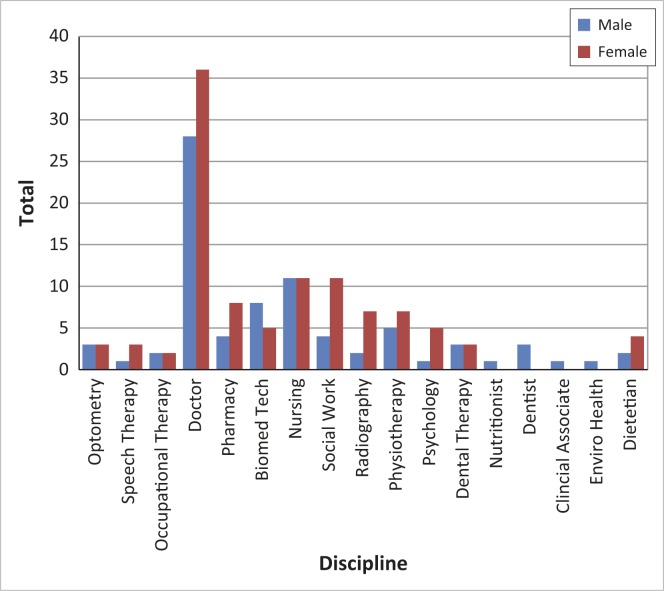
Breakdown of graduates by gender.

Currently 57% (106/185) of the graduates are women, and qualification by gender is presented in Figure 5. Slightly more women than men have qualified as doctors and pharmacists, whilst numbers are equal for nursing and occupational therapy. More men than women have trained as biomedical technologists, nutritionists, dentists and clinical associates.

## Discussion

The UYDF results are significant, particularly within the current SA context, and more so because of the rural origin of these students. With a pass rate of greater than 89% over a 15-year period and less than 10% of students being excluded from training institutions, these figures highlight the latent potential of rural scholars. These figures are also in sharp contrast to the national experience where, despite the number of black students at institutes of higher learning (IHL) rising from 30% in 1999 to 66% in 2010, this has not translated into an increased number of black graduates.

Cohort studies have shown that the completion rates of black African students at contact universities in the life sciences, mathematics and physical sciences is only about 33%, which is about half the completion rate of white students.^14^ Other scholarship programmes have had varied student success, with the Rural Education Access Program (REAP) reporting 57% – 66% student completion rates of the 131 students supported in 2002.^15^

**FIGURE 6:**
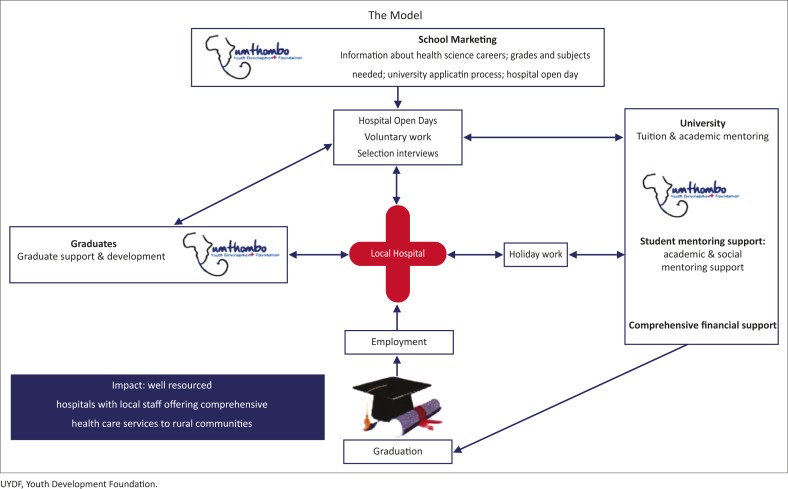
The UYDF model for student selection and support.

The success of the UYDF model may be related to several factors, including the following: (a) local hospital participation; (b) academic and peer mentoring; (c) social support; and (d) the reintegration and support of graduates into the hospitals once they have completed their training. These aspects are depicted in Figure 6.

The local DH is at the heart of the UYDF model, as the scholarship scheme started as a response to the need for staff at a rural DH. To ensure that the scheme is responsive to the needs of the hospital, the hospital selection committee is responsible for the promotion of a career in health through open days, and selection of health science students according to hospital priorities. UYDF students are also expected to do four weeks of experiential holiday work at the local hospital each year. This enables students to consolidate their theoretical knowledge in a practical way, and to apply their knowledge at a DH level, work alongside local HCPs and strengthen relationships with one another, with management at the hospital and with community members. This holiday work also prepares them for working at a DH as they understand the local conditions, challenges and resource constraints at such hospitals as well as what is expected of them as HCPs. Career-specific work experience is recognised as an important motivating influence for students at IHL and can contribute to students persisting and achieving academic success at IHL.^14^ Experiential holiday work is an integral aspect of the UYDF model.

UYDF is responsible for facilitating the academic and peer mentoring and the social support at university. Rural students often feel alienated when they first attend IHL, and this contributes to their social isolation and inability to access social and academic support.^16^ The UYDF provides a ‘family’ of other rural origin students who help each other to adapt to the challenges of university and city life. TM, a UYDF graduate, put it this way: ‘I would mentor new students. Not teaching them maths and physics, but I would mentor them in terms of social life, and how to handle the situation, knowing their background. So it was easier for the new guys to adapt in that environment, because I was there.’

Social and academic engagement has been identified by Tinto as critical to student success at IHL.^17^ Academic mentoring and support is provided by UYDF mentors who meet regularly with UYDF students to review their progress and ensure that academic and social issues are being addressed. This accountability encourages and supports students to find solutions to any challenges they might face at university. The academic mentoring also communicates a belief that students have the potential to succeed and that they belong at an IHL.

Other studies have shown that when students believe that they have the ability to succeed and that they belong or deserve to be at an IHL this contributes to their success.^18^ Laude at the University of Texas found that the introduction of small classes, mentorship and support which communicated to students that they had the ability to succeed and that they belong at university, influenced success of students who traditionally failed.^19^ The academic and peer mentoring as well as social support facilitated by UYDF is considered key to students’ success, as it enables them to identify and overcome academic and social challenges in order to succeed at IHL.

Comprehensive financial support is also an important component of the support provided by UYDF, which may assist in the success of the scheme by allowing students to focus more on the academic challenges at university. Inadequate finances have been identified as an important reason why students in SA fail at IHL.^16,20^ A criticism of the current NSFAS (National Student Financial Aid Scheme) funding model is that students only receive partial financial support, which covers fees and residence with only a small food and book allowance. This NSFAS model is based on the assumption that parents should make a family contribution towards these costs. However, for many rural students the family contribution is not forthcoming, and hunger and their limited access to the necessary resources distract from academic work and may contribute to their high failure rate. The Rural Education Access Programme, which supports many disadvantaged students, has recommended a review of the current funding model for NSFAS to ensure comprehensive funding for financially needy students at IHL.^16^

### Recommendation

The UYDF provides a model which could be replicated in other parts of SA. However, further studies are needed to identify and understand the key aspects of the UYDF model and whether or not this model can be taken to scale.

## Conclusion

Results from the last 15 years of the UYDF are significant and highlight that rural students can succeed at university with appropriate support. Whilst numbers are still small, all graduates have spent time working at rural hospitals, thus helping to ensure services are provided at these facilities. With 71% of those who have completed their work-back obligation having remained in rural sites, these numbers are encouraging and support data from other countries that rural origin students will return to work in rural areas.

As most of the current literature around the recruitment and training of rural origin students to provide services in rural areas is based on Australian and Canadian studies, this study adds to the body of literature by showing that even in developing countries, strategies to identify and support rural origin students to train as healthcare providers can contribute to the staffing of rural healthcare facilities.
